# Improving Diagnostics with Deep Forest Applied to Electronic Health Records

**DOI:** 10.3390/s23146571

**Published:** 2023-07-21

**Authors:** Atieh Khodadadi, Nima Ghanbari Bousejin, Soheila Molaei, Vinod Kumar Chauhan, Tingting Zhu, David A. Clifton

**Affiliations:** 1 Institute of Applied Informatics and Formal Description Methods, Karlsruhe Institute of Technology, 76133 Karlsruhe, Germany; 2 Independent Researcher, Tehran 009821, Iran; bousejin@gmail.com; 3 Department of Engineering Science, University of Oxford, Oxford OX1 3AZ, UK; vinod.kumar@eng.ox.ac.uk (V.K.C.); tingting.zhu@eng.ox.ac.uk (T.Z.); david.clifton@eng.ox.ac.uk (D.A.C.); 4 Oxford-Suzhou Centre for Advanced Research (OSCAR), Suzhou 215123, China

**Keywords:** electronic health record, deep learning, intensive care unit, deep random forest, representation learning

## Abstract

An electronic health record (EHR) is a vital high-dimensional part of medical concepts. Discovering implicit correlations in the information of this data set and the research and informative aspects can improve the treatment and management process. The challenge of concern is the data sources’ limitations in finding a stable model to relate medical concepts and use these existing connections. This paper presents Patient Forest, a novel end-to-end approach for learning patient representations from tree-structured data for readmission and mortality prediction tasks. By leveraging statistical features, the proposed model is able to provide an accurate and reliable classifier for predicting readmission and mortality. Experiments on MIMIC-III and eICU datasets demonstrate Patient Forest outperforms existing machine learning models, especially when the training data are limited. Additionally, a qualitative evaluation of Patient Forest is conducted by visualising the learnt representations in 2D space using the t-SNE, which further confirms the effectiveness of the proposed model in learning EHR representations.

## 1. Introduction

Medical and therapeutic techniques have substantially benefited from the collection of health data and the use of such data in the field of data science [1,2,3]. EHRs are one of these enormous sources of data, helpful for a variety of predictive tasks in medical applications [4]. EHRs hold a patient’s demographics, medical history, vital signs, laboratory tests, recommended medicine, diagnosis, and clinical outcomes during an interaction [5,6]. EHR databases may contain several patient visits, establishing a longitudinal patient record that can be used to aim the treatment process, such as disease prediction, mortality prediction, and enhancing the efficacy of the therapeutic process.

Initially, EHR systems were intended to manage the basic administrative functions of hospitals, permitting the use of regulated terminology and labelling schemes. Numerous labelling schemes exist, including ICD (International Statistical Classification of Diseases) codes for diagnostic [7,8,9,10], CPT (Current Procedural Terminology) codes for procedures [11,12,13], and LOINC (Logical Observation Identifiers Names and Codes) for laboratories [14,15], ATC (Anatomical Therapeutic Chemical) for drug [16,17], and RxNorm for medication [12]. The various labelling techniques produce standard datasets for varied specialisations. As the EHR system develops, the volume of EHR data increases annually, and several studies have been conducted on the secondary use of these data.

EHRs offer numerous benefits, including improved patient care, increased efficiency, and reduced healthcare costs [18]. Regardless of the potential for EHRs in various applications, their effective usage is hindered by data-specific restrictions [6], such as high missingness and irregular sampling [19,20,21], as well as imbalanced classes due to uneven prevalence of illnesses [22]. Therefore, it is important to address these limitations in order to fully realise the potential of EHRs.

Previous work on learning EHRs representations has mainly focused on developing methods for data integration, such as ontology-based mapping or semantic matching, which often require significant manual effort and are limited in their effectiveness in combining different data types. To address these limitations, our proposed approach leverages the latest advancements in deep learning and representation learning to create a more unified representation of EHR data. Our method utilises neural networks to learn a compact and meaningful representation of EHR data, which can be used for a variety of disease prediction tasks. Additionally, our approach can combine different data types effectively, such as demographic information, laboratory test results, and imaging studies, into a single, unified representation. This enables us to capture the complex relationships between different data elements and provides a more complete picture of the patient’s health status.

Classic machine learning approaches, such as Support Vector Machine (SVM) [23] and Random Forest (RF) [24], have been used previously to process large EHR datasets. However, these methods are limited in their ability to capture the complex patterns in the underlying data. In comparison, deep learning approaches based on deep data feature analysis are capable of producing efficient and reliable analytical outcomes, particularly in the real-world context of enormous data volumes. While deep learning-based models have shown promise in improving patient outcome prediction, they often require large amounts of data and computational power, which may not be available in all settings. Compared to deep learning models, Patient Forest offers several advantages. By using an ensemble of decision trees, Patient Forest is able to perform well even with smaller datasets and less computational power, which can be important in clinical settings.

In this work, we propose a novel approach, called Patient Forest, to learning EHRs representations based on the cascade deep forest method [25]. Our Patient Forest technique incorporates statistical features to create a more accurate classifier for predicting readmission and mortality. Our approach seeks to automate the process of data integration and generate representations that capture the complex patterns in the underlying data. Moreover, our technique performs a brief preprocessing in order to optimise the final performance. RF is a widely used technique for assessing data with a large dimension [26], and by utilising its random attribute, generating several forests, and getting multiple outcomes simultaneously in each layer of the deep network, our approach is able to enhance the performance of the predictions.

The main contributions of this paper include:

- We propose Patient Forest, a machine-learned approach for predicting patient outcomes, that incorporates statistical features to learn EHR representations.
- We conduct an evaluation of our proposed method on two large-scale EHR datasets and demonstrate its effectiveness in predicting readmission and mortality outcomes.
- We compare the performance of Patient Forest with strong machine learning baselines to reveal the effectiveness of our approach to improve patient care and reduce healthcare costs.

## 2. Related Work

In this section, we present a summary of related works in representation learning for EHR applications.

### 2.1. Vector-Based Methods

One of the learning models that represents patient information on this basis is a fully connected Deep Neural Network (DNN). Futoma et al. [27] evaluated various models’ propensity to forecast hospital readmissions using data from a large EHR database. The outcome demonstrates DNN outperforms other approaches that have previously been used to solve this issue in terms of prediction performance. The study given in [28] employed a deep generative learning model to overcome the problem of insufficient data using MRI pictures efficiently by learning and categorising tumour locations from MRI images. The search by Zheng et al. [29] for suicide ideation, behaviour, or death prediction in the literature was based on the health records of patients who had visited a Berkshire Health System hospital. Multiple machine learning and deep learning methodologies are employed in EHRs to classify the severity of patients in [30]. The experimental findings indicate DNN performed exceptionally well. In the type II diabetes disease prediction [31], a deep learning neural network architecture model was adopted. All these studies demonstrated the DNN can be utilised for EHR data analysis and diagnosis. Despite this, the majority of recent research has considered this architecture to be the classic way [32].

Autoencoders are vector-based, unsupervised deep learning models, which are an efficient dimensionality reduction technique with promising performance for the deep representation of medical data [33]. Autoencoders have also been effectively applied to datasets comprising massive collections of electronic health records, where they are very adept at handling missing data [34]. A comparison study by Sadati et al. [35] emphasised the effectiveness of several types of autoencoders for electronic health record-based data sets. Combining a recurrent autoencoder with two GANs, Lee et al. [36] suggested sequential electronic health records with a dual adversarial autoencoder (DAAE). Biswal et al. [37] synthesised sequences of discrete EHR encounters and encounter features using a variational autoencoder. Very recently, in [38], for adverse drug event preventability, a model of dual autoencoders was explored in EHRs. Wang et al. [39] compared the model with autoencoder features to traditional models, which could show a reasonable result.

Convolutional Neural Networks (CNNs) are a further vector-based technique. EHR research [40] focuses on capturing the local temporal dependence of these data, which are then used to predict multiple diseases and for other related tasks. Wang et al. [41] adopted a CNN learning with 1929 features for the classification of 1099 international diseases. Researchers in [42] aimed to develop a convolutional neural network model for the prediction of the risk of advanced nonmelanoma skin cancer (NMSC) in Taiwanese adults. In an intriguing study [43], CNN was applied over electronic health records to determine the top 20 lung-cancer-related indicators in order to avoid radiation exposure and costs. CNN has shown its superior ability to measure patient similarity. However, the traditional CNN architecture could not properly exploit the temporal and contextual information of EHRs for disease prediction. Consequently, it is increasingly difficult to represent the timing and substance of EHR data concurrently [44].

Natural language processing was the original inspiration for word2vec [45], which was developed to learn word embeddings from large-scale text resources. In [46], the authors pursue the word2vec technique to train a two-layer neural network to improve clinical application prediction accuracy relative to baselines. Choi et al. [47] applied skip-gram to longitudinal EHR data to learn low-dimensional representations of medical concepts. To improve the performance of a convolutional neural network for patient phenotyping, Yang et al. [48] explored a model that combines token-level and sentence-level inputs. Similarly, in [49], clinical text was employed to expect clinical notions. Steinberg et al. [50] proposed a novel analogy of language modelling on discretised clinical time-series data. However, these techniques do not explicitly model dynamic temporal information or address the challenges of heterogeneous data sources [51].

### 2.2. Temporal Matrix-Based Methods

Lee and Seu [52] presented Non-Negative Matrix Factorisation (NMF) as a method for discovering a collection of basic functions for expressing non-negative data. This matrix pertains to electronic health records, which generate a matrix with a time dimension and a clinical event dimension. Bioinformatics has extensively used NMF for clustering sources of variation [53,54,55]. There are other efforts to use NMF or its variants in the depiction of patient data in EHRs. In [56], disease trajectories are analysed using NMF to extract multi-morbidity patterns from a huge data collection of electronic health records. Zhao et al. [57] suggested that the NMF identifies relationships between genetic variants and disease phenotypes. In a recent study [58], NMF was used to examine the symptoms of covid and predict long-term infection. Controlling the degree to which the representation is sparse is difficult since sparseness is a side effect of the NMF algorithm [59]. The huge number of various diagnosis codes is an additional obstacle that results in a combinatorial explosion of the number of possible diseases, many of which are unique to a single patient [60].

### 2.3. Graph-Based Methods

The graph technique can be expressed using the EHR by using nodes to represent medical events and edges between the nodes to highlight the temporal links among clinical events. One emerging method of deep learning on graph-structured data is Graph Neural Networks (GNNs) [61]. GNNs can infer the missing information, leading to a representation that is more explicable [62]. The hierarchical relationships in EHRs were captured using GNN, as described in reference [63,64]. In [65], GNN reflected the links between drugs, side effects, diagnosis, associated treatments, and test results. For instance, Park et al. [66] suggested a knowledge graph-based question answering with EHR. Research [67] introduced an EHR-oriented knowledge graph system to efficiently utilise non-used information buried in EHRs. In EHRs, it is typical for spurious edges to be included and for other edges to be absent. Even though the observed graph is clean, it may contravene the properties of GNNs because it is not jointly optimised with them. These flaws in the observed graph may precipitously degrade the performance of GNNs [68].

### 2.4. Sequence-Based Methods

Sequence-based patient representation turns EHR data into a temporally ordered sequence of clinical events for use in prediction. A recurrent neural network (RNN) is a neural network that includes the GRU and LSTM networks as specific cases, according to Sherstinsky’s study [69]. RNNs are widely used in patient representation research that focuses on combinations or sequences of clinical codes [62]. The research included aid in early diagnosis [70,71] and disease prediction [72,73,74,75,76,77,78,79]. Recently, Gupta et al. [80] adopted a general LSTM network architecture to make improved predictions of BMI and obesity. Ref. [81] examined the performance of various deep neural network architectures, including LSTM, in scenarios involving clinical factors and chest X-ray radiology reports, revealing that the recommended BiLSTM model outperforms other DNN baseline models. RNN is frequently stated without context or rationale. In addition, training equations are frequently removed entirely; therefore, partial descriptions or missing formulas in RNN may result in its inefficiency [69].

### 2.5. Tensor-Based Methods

Tensor-based methods apply an n-dimensional tensor to represent patient information. The multi-dimensional and high level of tensor factors in EHR data make complex relationships understandable and interpretable [82]. Zhao et al. [83] identified previously unknown cardiovascular characteristics using a modified non-negative tensor-factorisation technique. Afshar et al. [84] implemented temporal and static tensor factorisation to extract clinically significant characteristics. Hernandez et al. [85] used a novel tensor-based dimensionality reduction method to predict the onset of haemodynamic decompensation.

## 3. Methodology

**Figure 1:**
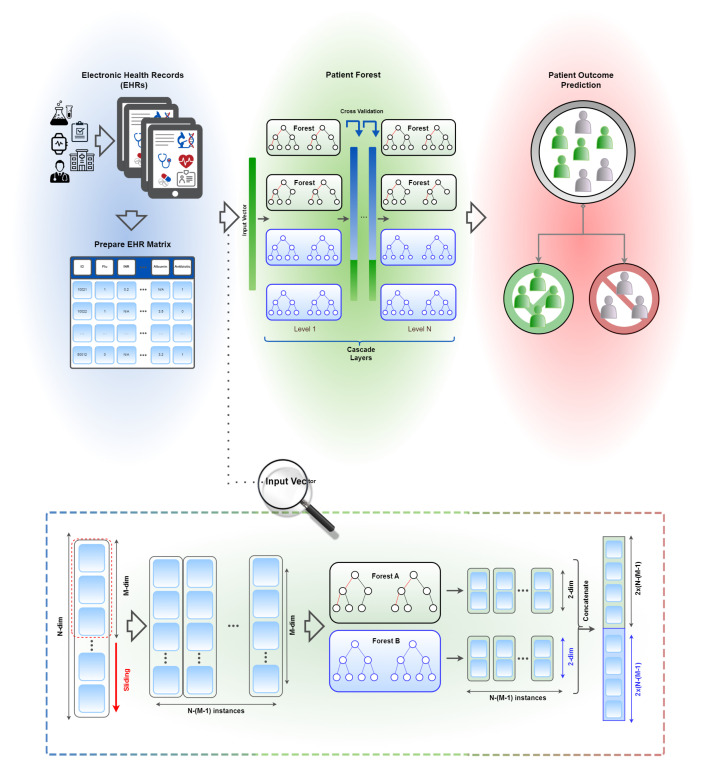
Schematic representation of Patient Forest, illustrating the process of patient outcome prediction based on learnt EHR representations from a prepared EHR matrix.

In the following, the specifics of the proposed approach will be presented. We frame the patient outcome prediction task as a classification problem. Patient Forest uses a prepared EHR matrix to learn EHR representations by using an ensemble of decision trees. These representations then may be retrieved and used for downstream patient outcome prediction tasks. Figure 1 depicts a summary of the model. In the subsequent sections, we explain the implementation details and experimental setup.

### 3.1. Patient Forest

We are provided with a set of *N* encounters, $X=\{\vec{x_{1}},\vec{x_{2}},\ldots ,\vec{x_{N}}\}$, as input, where $\vec{x_{i}}\in R^{F}$ represents encounter *i* features. We feed encounters to the gcForest model [25] in order to learn EHR representations. The learnt EHR representations are then used for downstream patient outcome prediction tasks.

The gcForest model is made up of two distinct modules: multi-grained scanning and cascade forest. The multi-grained scanning module is responsible for generating a set of diversified features from input data by using multiple layers of sliding windows (convolutional filters) of different sizes, which results in a set of sub-sampled feature maps, each capturing different aspects of the input data at different granularities. The output of this module is then fed to the cascade forest module.

The cascade forest module is made up of multiple levels of random forests. Each level takes as input the output of the previous level and further refines the extracted features. The final output of the cascade forest is a set of predictions for the input data.

During training, the gcForest model learns the input features by optimising the parameters of the convolutional filters and random forests using a backpropagation algorithm. These learned features are then used to make predictions on new, unseen data. For the training objective, we use a standard binary cross-entropy (BCE) loss between the target and predicted labels.

(1) $$L=-\frac{1}{N}\sum_{i=1}^{N}\left(\hat{y_{i}}\log\left(y_{i}\right)+\left(1-\hat{y_{i}}\right)\log\left(1-y_{i}\right)\right)$$

where $\hat{y_{i}}$ is the network’s predicted label, and $y_{i}$ is the ground-truth label.

### 3.2. Datasets and Preprocessing

#### 3.2.1. Datasets

Our proposed model examined, using the eICU [86] and MIMIC-III [87] datasets, both of which are large-scale electronic health record (EHR) dataset collections and are accessible through the PhysioNet repository [88].

eICU. Philips Healthcare has created the eICU Program, a telemedicine system that utilises these data to aid in the treatment of critically sick patients. The eICU Collaborative Research Database is a multi-centre intensive care unit (ICU) database providing high-resolution data for over 200,000 admissions between 2014 and 2015, to one of 335 units at 208 US hospital institutions. The de-identified database contains information such as vital sign readings, care plan paperwork, sickness severity measurements, diagnostics, and treatments [86].

MIMIC-III. Medical Information Mart for Intensive Care III (MIMIC-III) is a big, single-centre database including information on Beth Israel Deaconess Medical Center (BIDMC) in the United States from 2001 to 2012. Data comprises vital signs, medicines, laboratory measures, observations and comments documented by care providers, fluid balance, procedure codes, diagnostic codes, imaging reports, hospital length of stay, and survival data [87].

Readmission rates and death rates are the primary outcomes that catch our attention for the purpose of this article.

#### 3.2.2. Preprocessing

The EHR representations on MIMIC and eICU datasets are learned by following the preprocessing suggested in [89]. To prep the datasets, we removed encounters that were shorter than 24 h and eliminated duplicate codes (e.g., repeated medication administration). Lab results were also excluded as their values can change during ICU stays (e.g., blood pH level). These steps resulted in 50,391 and 41,026 patients in the MIMIC and eICU datasets, respectively. Throughout the paper, we focus on a single encounter and do not consider the time-series nature of EHRs. See Table 1 for the statistical details of the two datasets.

### 3.3. Baselines and Tasks

#### 3.3.1. Baselines

To assess our model’s performance in prediction tasks, we evaluate it against different baselines, including a Random Forest, Multi-Layer Perceptron, Logistic Regression, Support Vector Machine, Naïve Bayes, Classification and Regression Trees, and K-Nearest Neighbours.

- Random Forest (RF): Random Forest is a classifier comprising decision trees, where each tree at input x casts one vote for the most popular class [90].
- Multi-Layer Perceptron (MLP): Multi-Layer Perceptron is a type of artificial neural network consisting of multiple layers of neurons that are connected to each other and used for supervised learning tasks such as classification [91].
- Logistic Regression (LR): Logistic Regression is a machine learning algorithm used for predicting binary outcomes given a set of features [92].
- Support Vector Machines (SVM): Support Vector Machines are a type of supervised learning algorithm used for finding the line that maximises the minimum distance to the line [93].
- Naive Bayes (NB): Naive Bayes is a probabilistic classifier based on the assumption that all features are conditionally independent of each other given a class label [94].
- Classification and Regression Trees (CART): Classification and Regression Trees are a type of decision tree algorithm used for classification and regression problems that employ past data to generate decision trees, which are then used to categorise fresh data [95].
- K-Nearest Neighbours (K-NN): K-Nearest Neighbours is a non-parametric algorithm used for classification by identifying the closest k-neighbours to an observation and then assigning it a class label [96].

#### 3.3.2. Tasks

The purpose of this study was to compare Patient Forest and baseline models in their ability to predict two primary tasks: readmission prediction and mortality prediction.

- Readmission Prediction: The models were trained to extract visit representations from an encounter record in order to predict whether a patient will be readmitted to the ICU within the same hospital stay. This task was evaluated using only the eICU dataset.
- Mortality Prediction: The models were trained to extract visit representations from an encounter record in order to forecast patient mortality upon ICU admission. This task was assessed using both the MIMIC and eICU datasets.

### 3.4. Training and Evaluation

The gcForest model used in our experiments had a cascade structure comprising 4 completely random tree forests and 4 random forests, with 500 trees in each forest. Class vector generation was achieved using three-fold cross-validation. The number of cascade levels was automatically determined by splitting the training set into a growing set and an estimating set. The cascade was retrained after determining the estimated number of levels. Multi-grained scanning was performed using three different window sizes for feature windows. Moreover, 80% of the training data was used for the growing set and 20% for the estimating set.

We used two metrics to measure how accurately our patient outcome prediction tasks were performed: the Area Under the Precision-Recall Curve (AUPRC) [97] and the Area Under the Receiver Operating Characteristic Curve (AUROC) [98]. These metrics were calculated on the test set, which had the same class distribution as the actual data. AUPRC is sensitive to the proportion of positive outcomes, so the lowest possible value and the value of a random classifier would depend on the positive class rate [99]. AUROC is a measure of how well a classifier can separate positive and negative outcomes, regardless of the class distribution. The AUROC value of a random classifier is always 0.5.

We conducted 20 runs of training across three different train–test set splits (75%:25%, 50%:50%, and 30%:70%) and evaluated the performance of patient outcome prediction tasks across our baselines by measuring the AUPRC and AUROC on the test nodes of our method. To prevent data contamination, we used a patient-level split of the data, ensuring that each patient was included only in one split.

## 4. Experiments

**Figure 2:**
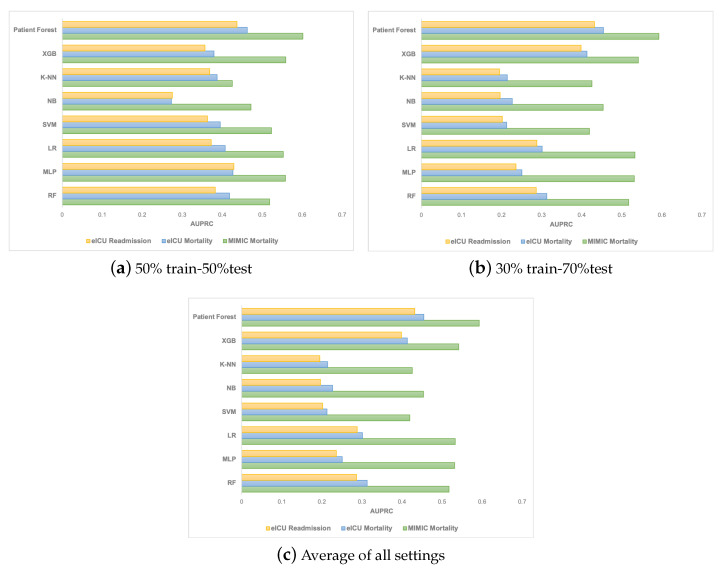
Readmission and mortality prediction performance on eICU and MIMIC in terms of AUPRC with different data splits: 50%:50% (**a**), 30%:70% (**b**), and average of all settings (**c**).

We compared the results of our Patient Forest model to several baseline models such as RF, MLP, LR, VM, NB, CART, and K-NN. The performance was evaluated using the AUPRC and AUROC metrics, and the results are reported in Table 2 and Table 3 for the 70%:30% data split and Figure 2 for the 50%:50% and 30%:70% data splits.

### Results

**Figure 3:**
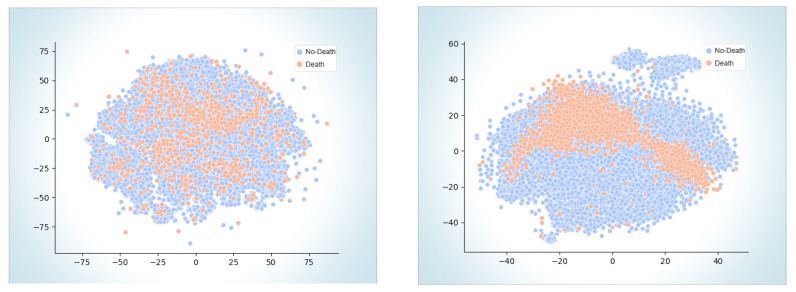
t-SNE embeddings of the patients in the MIMIC dataset based on raw (**left**) and learnt Patient Forest features (**right**).

- Predictive Performance: We compared the performance of Patient Forest with other baseline models, such as RF, MLP, LR, SVM, NB, CART, and K-NN. We used two metrics to evaluate the models: AUPRC and AUROC. AUPRC measures how well the model can identify the positive class (i.e., patients who died or were readmitted), while AUROC measures how well the model can separate positive and negative classes (i.e., patients who survived or were not readmitted). Higher values of both metrics mean better performance. We used two datasets: MIMIC and eICU, and three data splits: 75%:25%, 50%:50%, and 30%:70%. Table 2 and Table 3 show the results for AUPRC and AUROC using the 75%:25% split. Figure 2 shows the results for AUPRC using the other two splits. The results show that Patient Forest consistently outperformed all the baseline models on both metrics and both datasets.
  Using the 75%:25% train–test split, the Patient Forest model outperformed all the other models on both AUPRC and AUROC. It achieved 0.619 AUPRC and 0.8010 AUROC for MIMIC Mortality, 0.5732 AUPRC and 0.8637 AUROC for eICU Mortality, and 0.5952 AUPRC and 0.8690 AUROC for eICU Readmission. Using the 50%:50% split, the model still demonstrated superior performance with AUPRC of 0.6019, 0.4626, and 0.4372 in MIMIC Mortality, eICU Mortality, and eICU Readmission, in order. Even with a reduced percentage of training data (30%), our model still outperformed the other baselines with AUPRC of 0.5927, 0.4544, and 0.4317 in MIMIC Mortality, eICU Mortality, and eICU Readmission, respectively.
  These results demonstrate the robustness and generalizability of our model across different datasets and data splits. The superior performance of Patient Forest can be attributed to its ability to capture the heterogeneity and complexity of the patient data using a forest of patient-specific decision trees. By learning from the patient’s own history and features, our model can better predict the patient’s future outcomes than the models that use a single global classifier for all patients. Moreover, by aggregating the predictions of multiple trees, our model can reduce the variance and improve the stability of the results.
- Comparative Evaluation: To assess the effectiveness of Patient Forest and its learned representations, we plotted t-distributed stochastic neighbour embedding (t-SNE) plots [102] of the generated representations (Figure 3) for the MIMIC mortality prediction task. The different colours denote different patient classes. Our qualitative results demonstrate Patient Forest is able to learn representations that place patients with similar outcomes close to each other, indicating the model’s capability of accurate prediction of outcomes.

## 5. Discussion and Conclusions

We presented Patient Forest, a model which can learn EHR representations to predict mortality and readmission rates. Our proposed approach was trained and tested on two extensive EHR datasets and three benchmark tasks. We compared its performance with other prominent models and highlighted the benefits of our model. Furthermore, we did a qualitative assessment of Patient Forest by plotting the t-SNE of the embeddings for the targeted outcomes across two studied datasets. Results indicated that the learnt representations provide a 2-D projection that clearly reveals clustering.

This study has notable merits. Patient Forest can accurately learn EHR representations and surpass other strong models, especially when the training data is scarce, which is a common difficulty in healthcare domains [103]. We also validated our proposed model on two expansive EHR datasets. Our method can provide valuable insights and guidance for clinicians and patients to improve the quality of care and health outcomes.

Conversely, there are certain limitations to this study. We did not factor in the temporal properties of EHRs. Additionally, we only incorporated three primary tables from MIMIC and eICU datasets, such as diagnosis, laboratory results, and treatment tables. We intend to explore strategies to extend our work to time-series EHRs, which could be beneficial for learning representations of patient deterioration tasks over time and include more tables like demographics and procedures in upcoming works.

To summarise, our study reveals that Patient Forest is a viable model for predicting mortality and readmission rates. Furthermore, it should be explored for its potential in other areas such as disease stratification, diagnostics, and prognosis. Additionally, research should be done to determine the optimal hyperparameters of Patient Forest, as well as explore its integration with other AI models to enhance accuracy and performance. Last, the application of this model in a clinical setting must be evaluated to assess its utility for healthcare professionals.

## Figures and Tables

**Table 1:** Statistical characteristics of the datasets used to train and evaluate the model for both the mortality and readmission tasks. N/A stands for Not Applicable.

Dataset	MIMIC	eICU
Avg. # of diagnosis per visit	11.5	6.5
Avg. # of treatment per visit	4.5	5.0
# of Positives (Readmission)	N/A	7051
# of Positives (Mortality)	5377	2983
# of Patients	50,391	41,026

**Table 2:** Readmission and mortality prediction performance on eICU and MIMIC in terms of AUPRC using 75%:25% data split.

Models	MIMIC Mortality	eICU Mortality	eICU Readmission
RF [90]	0.5166	0.4843	0.4528
MLP [100]	0.5676	0.4549	0.4519
LR [92]	0.5863	0.4903	0.4628
SVM [101]	0.5602	0.5193	0.4612
NB [101]	0.4506	0.3532	0.3491
K-NN [96]	0.4575	0.4196	0.4016
XGB [96]	0.5187	0.4599	0.4481
Patient-Forest (ours)	0.619	0.5732	0.5952

**Table 3:** Readmission and mortality prediction performance on eICU and MIMIC in terms of AUROC using 75%:25% data split.

Models	MIMIC Mortality	eICU Mortality	eICU Readmission
RF [90]	0.7628	0.8392	0.8136
MLP [100]	0.7492	0.8163	0.8121
LR [92]	0.7732	0.8328	0.8192
SVM [101]	0.7463	0.8473	0.8213
NB [101]	0.6583	0.7628	0.7605
K-NN [96]	0.6538	0.7927	0.8001
XGB [96]	0.7719	0.8129	0.8083
Patient-Forest (ours)	0.8010	0.8637	0.8690

## Data Availability

Datasets are accessible through the PhysioNet repository [88] at https://www.physionet.org/.
